# Twelve tips for incorporating gamification into medical education

**DOI:** 10.15694/mep.2019.000216.1

**Published:** 2019-11-26

**Authors:** Shabnam Singhal, Josephine Hough, David Cripps

**Affiliations:** 1 University of Warwick

**Keywords:** Gamification, serious games, game-based learning, GBL, educational games, narrative, feedback

## Abstract

This article was migrated. The article was marked as recommended.

Gamification incorporates elements of gameplay into real-word activities and behaviours. These elements include progress mechanics, player development and narrative structure. Gamification is being increasingly used in an educational context as it has the ability to make learning fun, memorable and more effective. We recommend ways in which gamification can be effectively implemented in medical education, including common pitfalls and hurdles to avoid.

## Introduction

Gamification has been defined as “the craft of deriving all the fun and addicting elements found in games and applying them to real-world or productive activities” ( Chou, 2012). There has been growing evidence for gamification in a wider pedagogical context, with the concept beginning to gain traction within the medical education community ( Ahmed *et al.*, 2015). These “fun and addicting elements” include progress mechanics (e.g. leaderboards/badges/levels), narrative structure and immediate feedback. A recent systematic review reported moderate evidence for the use of serious games with a pedagogical purpose, as an adjunct to traditional methods, using the Medical Education Research Study Quality Instrument (MERSQI) scale ( Gorbanev *et al.*, 2018).

Gamification is sometimes included within game-based learning or serious games, although it stands as a distinct entity. Gamification applies gaming principles to the learning process, e.g. narrative and progress mechanics, to make it fun and addictive ( Ingwersen, 2017). Game-based learning uses a game to teach a particular skill or learning outcome e.g. adapting the game Name-that-tune to Name-that-murmur ( Pitt *et al.*, 2015). Similarly, serious games are games designed for purposes other than entertainment, such as training or behaviour change ( Connolly *et al.*, 2012). However gamification, game-based learning and serious games all aim to use games to make learning more engaging and motivating ( Ingwersen, 2017).

Over the past 20 years, medical education games, mobile applications, and virtual patient simulations (together termed “gamified training platforms”) for both preclinical and clinical medical education have been developed. These range from Fold it, an online puzzle centred around protein folding, to Nuclear Event Triage Challenge, whereby players have to triage patients during nuclear events ( McCoy *et al.*, 2016).

Gamification offers unique learning opportunities in its ability to allow learners to navigate complex systems through game processes ( Bochennek *et al.*, 2007). This approach demands active engagement of learners, can influence behaviour and provide motivation.

In this paper, we will consider evidence from theoretical, biological, sociological perspectives as well as the authors’ own experience. We offer 12 tips to harness the power of gamification in medical education.

## 1. Make learning fun and engaging

**Figure 1.**
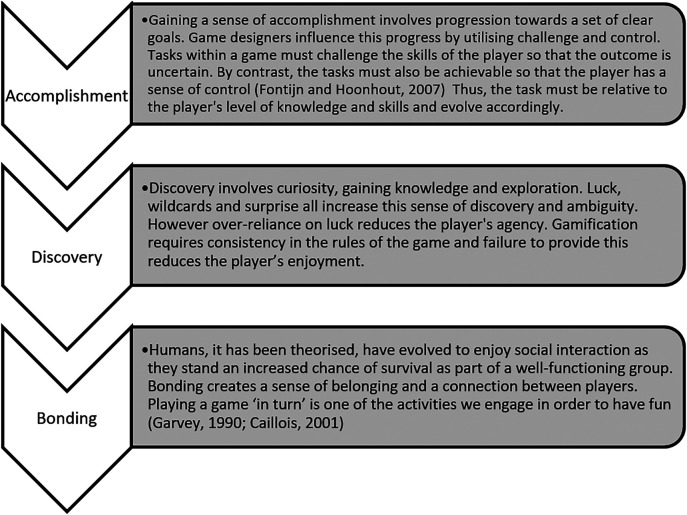
Adapted from Fontijn and Hoonhout (2007): Three core sources of fun and application to gamification

Fontijn and Hoonhout (2007) propose that fun is an evolutionary mechanism for rewarding certain behaviours, such as using skills and knowledge to increase chances of survival. By their nature, games demand the active engagement of learners. There is evidence that functional changes in the brain associated with learning occur best in this state of active engagement ( Friedlander *et al.*, 2011). Fontijn and Hoonhout (2007) define three core sources of fun: accomplishment, discovery and bonding ( Figure 1). Appealing to each of these three sources is a practical way of maximising fun in gamification.

Thus, for an educational game to be successful it must be fun, enjoyable and engaging.

## 2. Evoke intrinsic as well as extrinsic motivation

In a literal sense, to be motivated means ‘to want to do something’. Within medical education, learners need to be motivated in order to persevere with their studies and become competent clinicians. Intrinsic motivation refers to doing something because it is enjoyable or inherently interesting, whereas extrinsic motivation refers to doing something because it leads to a specific outcome ( Ryan and Deci, 2000). Gamification presents the danger of the ‘overjustification effect’ where additional external incentives can decrease a learner’s intrinsic motivation for the subject ( Meske *et al.*, 2017).

There is no clear conclusion within the literature as to whether gamification impacts more on learners who are intrinsically or extrinsically motivated. Buckley *et al.* (2016) report that gamification impacts learners in different ways, having a larger impact on learners who are intrinsically motivated. Studies have shown that in comparison to extrinsic motivation, intrinsic motivation leads to greater creativity, cognitive flexibility, conceptual learning as well as enhanced well-being ( Deci and Ryan, 2000). However, Banfield and Wilkerson (2014) report that extrinsic motivation, in the form of rewards achieved through competitive prizes, are an effective tool until intrinsic motivation can develop. Similarly, Muntean (2011) reports that gamification desires to combine intrinsic and extrinsic motivation, in order to increase motivation and engagement.

Gamification can therefore be a catalyst to provide extrinsic motivation in the form of short-term rewards to learners. However by sparking curiosity and interest in the core subject in an enjoyable way, gamification also has the power to reignite intrinsic motivation

## 3. Incorporate Progress Mechanics

Here we refer to mechanisms that allow a learner to see the progress they have made with their learning and earn ‘rewards’ as they progress. A basic example would be a series of short quizzes with a medical knowledge theme. Each learner has a ‘level’ (e.g. starting at level 1) and each time the learner scores a certain mark on a quiz they ‘level up’. Progression to higher levels could offer access to harder quizzes or other benefits. Other examples include badges, character upgrades and progress bars.

This is a concept that has been used to great effect in the videogame industry with highly addictive results. The long-term rewards for learning medicine are numerous, however short-term rewards can be scarce. Earning levels or badges through progress mechanics could act as a short-term reward to bridge this gap. Immediate rewards can strengthen learning and encourage engagement with learning processes at a neurobiological level ( Friedlander *et al.*, 2011). This can also help set clear goals and objectives by breaking subjects into smaller, more manageable topics. Therefore progress mechanics provide a powerful additional extrinsic motivator to engage with content.

## 4. Use a narrative structure

Whilst numerous definitions of the word narrative exist in the literature, here we discuss narrative as the story surrounding the development of a character as they take part in significant experiences. Humans use narratives as a way of understanding the world around them and recalling experiences ( Miller-Day and Hecht, 2013). Learner involvement in games can create narratives in which learning is embedded, which may later improve recall. This narrative synthesis can take place through immersion in a game environment where learners can take actions and subsequently see and respond to their consequences, connecting cause and effect in a meaningful way ( Mohan *et al.*, 2018).

For example, a game may present a learner with a simulated case with signs and symptoms of a specific condition and challenge the learner to choose a treatment from a list of options. Once the learner chooses their treatment, they are presented with the consequences of their choice - how the patient responds to the treatment. Thus, a short narrative around the treatment of the condition is created that is personal to the learner. This type of learning intervention has been shown to improve performance in validated simulator tests in the context of trauma triage ( Mohan *et al.*, 2018).

## 5. Make learning experiential

Experiential learning is a well-established concept in medical education and refers to learning occurring when experience is reflected upon ( Mann, 2011). Theoretically a game can offer a simulated experience that can be reflected upon to produce learning.

For example, instead of answering questions on the management of patients with abdominal pain, a game might put learners in the role of an emergency physician with a series of patients with abdominal pain and challenge them to come up with management plans. In this setting, the learner has the simulated experience of managing the patients. They can make mistakes in a safe low-stakes environment, whilst reflecting and learning from their experiences.

Whilst the value of this experience is highly variable depending on the quality of the game and could never replace real clinical experience, the costs (financial and otherwise) of providing this experience are much lower and allow it to be reproduced on a wider scale.

## 6. Effective Gameplayer feedback is key to development

A key motivator of human behaviour is a need to know how well we are doing, with the prospect of success being vital to increasing motivation and effort ( Atkinson, 1957). These factors increase when we anticipate success and decrease when the goal is deemed unlikely or impossible ( King, 1999). Feedback therefore engages learners in the learning process and aids in retention allowing the student to progress towards their final goal ( Chowdhury and Kalu, 2004; Kapp, 2012).

Feedback and subsequent learner development are therefore as essential in gameplay as they are in the experiential learning cycle in education ( Manolis *et al.*, 2013). Kapp (2012) states that the balance between learning and gameplay is a key factor of success.

The literature suggests there are some caveats to ensure effective feedback within gamification. For instance, unlike current traditional educational practices, feedback should be frequent and immediate or with shortened cycles. The more frequent and immediate the feedback is, the greater the learning effectiveness and engagement ( Kapp, 2012; Berkling and Thomas, 2013; Dicheva *et al.*, 2015). Clear and immediate feedback has also been shown to be integral for the flow state, which is noted to be a state of engagement and immersion in an activity and is an important element of gameplay ( Csikszentmihalyi, 1997). If feedback is not intrinsic to game mechanics, a post-game debrief would therefore be essential.

## 7. Ensure sustainability

The creation of gamification learning resources may take additional time and effort compared to traditional learning methods. This can present a significant barrier where educator resources are already spread thin ( Lee and Hammer, 2011). Therefore, gamification may work best for teaching that is delivered several times to subsequent groups of learners. Conversely it may be more difficult to incorporate into bespoke teaching sessions, such as bedside teaching.

It is important to consider how game elements could be disseminated and used by others. Including clear instructions can facilitate use by future educators or for independent use by learners. Also bear in mind the ways in which resources may be sustainably reused over several years. For example, in clinical cases use patient ages instead of dates of birth, especially in paediatric teaching. Paper resources can be laminated to prevent degradation or, to minimise environmental impact, use electronic resources. Online gamification resources can be easily replicated and distributed widely across geographical boundaries and time zones ( Choules, 2007). Many gamification repositories have banks of existing resources that can be reused and modified (e.g. Quizlet, Anki, Nearpod etc.).

## 8. Use technology and devices (when relevant)

The use of technology in gamification for medical education is not essential, however some report the process is more manageable by tracking accomplishments, point scoring and result aggregation ( Educause, 2011). Furthermore, technology is becoming increasingly important and prevalent as each generation of learners enters medical school with a higher degree of digital literacy. Thus, the use of gamified training platforms to enhance medical education is becoming a popular resource not only by tutors in the classroom or individually by learners, but by clinicians at all levels of training ( McCoy, Lewis and Dalton, 2016).

Video games set in virtual settings inspired by daily life present realistic challenges to players. This can result in more authentic learning, which is useful in real-life decision making ( Gresalfi and Barab, 2011). For example, utilising an A& E environment where learners would triage patients and make decisions based on clinical status. Utilising technology in this way may enhance the realism and relevance of a session ( Lombardi, 2007).

Technology enhanced gamification can also provide opportunities for improved engagement, problem solving and cooperative teamwork. These skills are integral to future health care delivery. Games are based on the interaction with other players in a social setting ( Akl *et al.*, 2010). Examples include TurningPoint (Turning Technologies, LLC), Bravo (C-3 software), and DecisionSim (Kynectiv, Inc.). Gameplay also has the potential to connect players to learning communities, allowing reflection and strategizing on a wider scale.

In the future, more emphasis is likely to be placed on the use of creative technology to produce collaborative multimedia projects within medical education ( Sandars and Schroter, 2007).

## 9. Repetition is central to learning

Although gamification can be used to introduce new concepts, it can also be used effectively in a revision setting to consolidate knowledge and improve recall. Medical students are required to memorise vast quantities of factual knowledge and repetition is key to reinforcing learning and retention ( Friedlander *et al.*, 2011). However repetition of topics may be difficult to achieve in an already compressed medical school curriculum, where multiple interests are vying for time and space ( Friedlander *et al.*, 2011). Gamification, which tends to be quick-fire, can cover a breadth of topics repeatedly in a time-efficient manner. Furthermore, testing learners is more efficient than repeated presentation of facts, as is encouraging active recall ( Augustin, 2014).

When using gamification, there may be a temptation to cover a diverse range of subjects, however repetition may be a more efficient way of reinforcing learning. Repetition of questions can cement uncertain knowledge of facts e.g. the steps of the asthma ‘treatment ladder’. Equally, repetition of themes allows learners to view the same topic from different angles, e.g. the side effects of each drug, contraindications, monitoring required. Returning to the same theme allows learners to build a multi-faceted view of the topic and aligns with a constructivist view of learning.

Spaced repetition (i.e. exposing learner to the material at increasing time intervals) can be particularly beneficial in improving long-term recall ( Augustin, 2014). This is built into certain gamification elements, such as Anki flashcards. Repetition also fuels the addictive elements of gamification by activating the reward and reinforcement neural pathways ( Friedlander *et al.*, 2011). This has been used to great effect in apps such as Candy Crush or Farmville ( Lee and Hammer, 2011).

## 10. Competition can improve motivation

A degree of competition can improve motivation and engagement with learning, especially amongst peers who consider each other academic equals ( Malone and Lepper, 1987; Janssen *et al.*, 2015). Competition in the classroom context may result in material rewards or simply the ‘prestige’ of winning. Competition can increase stress levels which has a variable effect on learning. Some degree of stress can be beneficial but excessive stress can impair learning ( Friedlander *et al.*, 2011). Some argue competition forces learners to focus on the goal instead of the learning process and that stress derived from competition has a greater negative effect than its benefits ( Cantador and Conde, 2010).

Managing stress levels when adding a competitive element to teaching is an important factor to consider. For example, a friendly low-stakes quiz could create a healthy amount of stress but answering quiz questions in front of a large audience could create a level of stress that becomes detrimental to learning. Thus, ideally a balance between competition and cooperation must be struck. Stress levels in competition can be mitigated to some degree with learners conferring amongst teams, as this dissipates the responsibility for failure.

## 11. Focus on Collaborative Learning

Despite the obvious links of gamification to competition, in order to mitigate stress levels we recommend a focus on collaboration when introducing gamification to the classroom. Gamification can act a catalyst for collaborative learning, where pairs or teams of learners can work together towards a shared goal or problem. This allows individuals to share their knowledge and abilities whilst distributing the responsibility amongst the group ( Laal and Ghodsi, 2012). Collaborative learning by working towards a common goal has been shown to lead to higher achievement, more supportive relationships and better self-esteem ( Laal and Ghodsi, 2012).

Many games and apps utilise these social elements to create a sense of community and maximise engagement ( Simões, Redondo and Vilas, 2013). There is a growing understanding of how social networks such as Twitter and Facebook can promote learner engagement and collaboration in medical education ( Cheston, Flickinger and Chisolm, 2013). Social cognitive theory emphasises this social aspect of education where learning occurs through the dynamic interaction of the individual with others ( Mann, 2011). Gamification can also improve learning outside the classroom by developing learning communities and a social support system, as well as promoting interpersonal skills ( Panitz, 1999). This is effective only if gamification is encouraged within a positive, supportive learning environment.

## 12. Integrate Diverse Player Types

Learners will come to gamification with a wide range of knowledge, skills, past experiences and personal attributes. Successful gamification in the classroom creates an environment that incorporates all of these. Learners vary in their preferences for how material is delivered and the learning strategies used ( Friedlander *et al.*, 2011).

Differentiated instruction, as described by Tomlinson *et al.* (2003) considers how the teacher can account for these variations in the classroom. Gamification firstly allows for the close monitoring and assessment of learner ability that is necessary for effective differentiated instruction. Furthermore, using gamification creates novelty and variety within the classroom that may appeal to the interests of particular learners.

When designing gamification elements for the classroom, consider how learners may work together to pool their diverse knowledge. These may also have broader appeal if calling upon a varied range of skills, such as speed, accuracy, visual interpretation, hand-eye co-ordination.

## Conclusion

In conclusion, we have combined theoretical frameworks with practical experience to provide an introductory guide to implementing gamification into the classroom. We have considered some of the barriers to gamification and the ways in which these can be overcome. Although each gameplay element presents an innovative new tool to engage and motivate students, consider only incorporating one or two gamification elements to avoid distraction from the core learning material. Gamification provides exciting new avenues that are largely yet to be explored and currently underutilised in medical education.

## Take Home Messages

• Making learning fun and experiential can improve learner motivation.
• Feedback and repetition can make learning more effective.
• Gamification is about collaboration, as well as competition.
• Ensure the gamification elements are reusable. Using technology may facilitate this.
• Consider implementing only one or two gamification elements at a time.

## Notes On Contributors

Dr Shabnam Singhal (ORCID ID https://orcid.org/0000-0003-1621-3521) completed her BMedSci (Hons) and BMBS (Hons) at the University of Nottingham. She is an Associate Fellow of the Higher Education Academy and has completed a Postgraduate Certificate in Medical Education from the University of Warwick. She has worked as a Clinical Teaching Fellow at South Warwickshire NHS Foundation Trust and is currently working as a junior doctor. She has an interest in Paediatrics and Medical Education.

Dr Josephine Hough (ORCID ID https://orcid.org/0000-0002-6092-549X) completed her MBChB at the University of Warwick following a 1st Class degree (Hons) in Human Biosciences at the University of Plymouth. She is an Associate Fellow of the Higher Education Academy and has attained a Postgraduate Certificate in Medical Education. She has previously worked as a Clinical Teaching Fellow at South Warwickshire NHS Foundation Trust. Currently she is working as a Junior doctor, soon to commence General Practice training. She has an interest in General Practice, Dermatology and Medical Education.

Dr David Cripps (ORCID ID https://orcid.org/0000-0003-1033-0858) completed his MBChB at The University of Manchester. He is an associate fellow of the Higher Education Academy and works as a Clinical Teaching Fellow at South Warwickshire NHS Foundation Trust. He has an interest in simulation and games in medical education.

## Declarations

The author has declared that there are no conflicts of interest.

## Ethics Statement

This research did not require Ethics Board approval because it did not involve human or animal subjects.

## External Funding

This article has not had any External Funding
