# Supplementary material for: Twelve tips for incorporating gamification into medical education
Source: MedEdPublish (2016). 2019 Nov 26;8:216. [Version 1] doi: 10.15694/mep.2019.000216.1 (PMC10712530; doi:10.15694/mep.2019.000216.1)
Supplement: Peer review history

# Reviewer report: Reviewer response for version 1

**DOI:** 10.21956/mep.19940.r30268

**Published:** 2020-01-17

**Authors:** Mary Dankbaar

**Affiliations:** 1 Erasmus University Medical Centre

This review has been migrated. The reviewer awarded 4 stars out of 5 Very interesting and well researched summary of main game characteristics that can be used in addition to regular learning activities (gamification). I agree with the authors that the number of gamification elements should be limited to one or two to keep the focus on the learning material and main message. Adding a few examples from teaching practice would make this paper more appealing for teaches.

Reviewer Expertise:

NA

No decision status is available

# Reviewer report: Reviewer response for version 1

**DOI:** 10.21956/mep.19940.r30266

**Published:** 2019-12-04

**Authors:** Trudie Roberts

**Affiliations:** 1 Leeds Institute of Medical Education

This review has been migrated. The reviewer awarded 3 stars out of 5 An interesting and timely paper and tips.Like others I would have appreciated it if the authors had then used their own tips in a description of how they have developed gamification in their own teaching

Reviewer Expertise:

NA

No decision status is available

# Reviewer report: Reviewer response for version 1

**DOI:** 10.21956/mep.19940.r30267

**Published:** 2019-11-29

**Authors:** Simonetta Ausoni

**Affiliations:** 1 Department of Biomedical Sciences- University of Padova- School of Medicine

This review has been migrated. The reviewer awarded 3 stars out of 5 I found the reading of this article interesting and stimulating. It would have been very useful to have concrete examples, since the authors have theoretical and practical experience on the subject. Sometimes the resistance of teachers arises precisely from the fact that they fail to see the improvement in teaching provided by new strategies, whereas they do see a lot of work to keep them on their feet. Therefore, although it is clear to readers that this is not presented as a research paper, some concrete examples and students’ feedback would have reinforced the article’s message.

Reviewer Expertise:

NA

No decision status is available

# Reviewer report: Reviewer response for version 1

**DOI:** 10.21956/mep.19940.r30264

**Published:** 2019-11-27

**Authors:** P Ravi Shankar

**Affiliations:** 1 American International Medical University

This review has been migrated. The reviewer awarded 4 stars out of 5 This is an interesting and well-written manuscript. Gamification is being increasingly used in medical education. The twelve tips provided by the authors seem practical and well-researched. I was first introduced to gamification in 2001 during a mass casualty training exercise conducted by the World Health Organization. We had used crosswords in pharmacology at the Manipal College of Medical Sciences in Pokhara. Recently I along with colleagues from the United Kingdom had used Braincept, a card-based role-playing game in revising the pharmacology of the respiratory system. I am sure the article and the tips will be very useful for educators planning to introduce games in the undergraduate curricula. I do agree with the authors that it is more prudent to proceed slowly and not introduce more than two or three games a semester.

Reviewer Expertise:

NA

No decision status is available

# Reviewer report: Reviewer response for version 1

**DOI:** 10.21956/mep.19940.r30269

**Published:** 2019-11-27

**Authors:** Junichi Tanaka

**Affiliations:** 1 tohoku university

This review has been migrated. The reviewer awarded 4 stars out of 5 This is a very interesting paper.But why do you think "consider implementing only one or two gamification elements at a time"?I think that by incorporating multiple elements well, we can establish gamification and promote learning.Just do a team-style quiz and it already includes elements of collaboration and competition. I think we can add other elements such as level up to make it more motivating.

Reviewer Expertise:

NA

No decision status is available

# Reviewer report: Reviewer response for version 1

**DOI:** 10.21956/mep.19940.r30265

**Published:** 2019-11-27

**Authors:** Alexander Woywodt

**Affiliations:** 1 Lancashire Teaching Hospitals NHS Foundation Trust

This review has been migrated. The reviewer awarded 4 stars out of 5 This is an interesting and well written paper on a very interesting topic. We recently discussed a gaming approach in our own department and I welcomed the opportunity to read a paper on this topic. I understand all the tips and they do make sense to me but I found the paper a little dry to read. I think I would have liked some more concrete and practical examples. This would help me to better understand this approach and its opportunities as well as limitations.

Reviewer Expertise:

NA

No decision status is available
